# Coincidental Occurrence of Hepatocellular Carcinoma and Cholangiocarcinoma (Collision Tumors) After Liver Transplantation: A Case Report

**DOI:** 10.5812/hepatmon.5871

**Published:** 2012-10-28

**Authors:** Waleed Al Hamoudi, Hatem Khalaf, Naglaa Allam, Mohammed Al Sebayel

**Affiliations:** 1 Gastroenterology Unit, King Saud University, Riyadh, Saudi Arabia; 2 Department of Liver Transplantation and Hepatobiliary-Pancreatic Surgery, King Faisal Specialist Hospital and Research Center, Riyadh, Saudi Arabia

**Keywords:** Hepatic Lesion, Biopsy, Recurrence, CA19-9 Antigen

## Abstract

Coincidental occurrence of hepatocellular carcinoma (HCC) and cholangiocarcinoma, known as “collision tumors”, within a cirrhotic liver is rare. Herein, we report a case of liver transplantation (LT) in a patient with such collision tumors. Our patient was a 56-year-old woman with hepatitis C virus-related cirrhosis and 2 focal hepatic lesions, measuring 1.5 and 3 cm, in the liver segments 8 and 5, respectively. The lesion on segment 8 showed the typical radiological characteristics of HCC; however, the lesion in segment 5 showed an atypical vascular pattern and was closely associated with the inferior vena cava. Serum alpha-fetoprotein level was normal and serum carbohydrate antigen 19-9 (CA19-9) level was slightly elevated (63 U/mL); the extrahepatic spread of HCC was ruled out. The patient underwent an uneventful deceased-donor LT. Histopathological examination of the explant confirmed that the lesion on segment 8 was an HCC, but surprisingly, the lesion on segment 5 was found to be a cholangiocarcinoma. Six months after LT, the serum CA19-9 level was markedly elevated (255 U/mL), and the patient began experiencing abdominal pain. Magnetic resonance imaging showed enlarged hilar and paraaortic lymph nodes that were suggestive of metastases; histopathological analysis using ultrasound (US)-guided biopsy confirmed recurrent cholangiocarcinoma. Unfortunately, the patient died because of tumor recurrence 9 months after LT.

Collision tumor resulting from the co-existence HCC and cholangiocarcinoma in a cirrhotic liver is rare and has a negative impact on the outcome of LT. Atypical vascular pattern and elevated serum CA19-9 levels are suggestive of such tumors; patients with these findings should undergo a targeted biopsy to rule out the coincidental occurrence of HCC and cholangiocarcinoma.

## 1. Background

Focal hepatic lesions in cirrhotic livers are common and suggest the presence hepatocellular carcinoma (HCC). Appropriate diagnosis of these lesions is crucial in choosing appropriate treatment strategies, ranging from liver transplantation (LT) to regular follow-ups (1). Recent advances in imaging techniques, including contrast-enhanced magnetic resonance imaging (MRI) can help accurately characterize most focal hepatic lesions; however, the risk of false diagnosis should always be considered, particularly in cases in which cholangiocarcinoma is suspected (2-4). Guided biopsy of the suspected lesions has very high sensitivity and almost absolute specificity; however, there is an underlying risk of malignant seeding after the procedure (5). Coincidental occurrence of HCC and cholangiocarcinoma, known as collision tumors, within a cirrhotic liver is rare. Herein, we report a case of LT in a patient with such collision tumors.

## 2. Case Report

**Figure 1:**
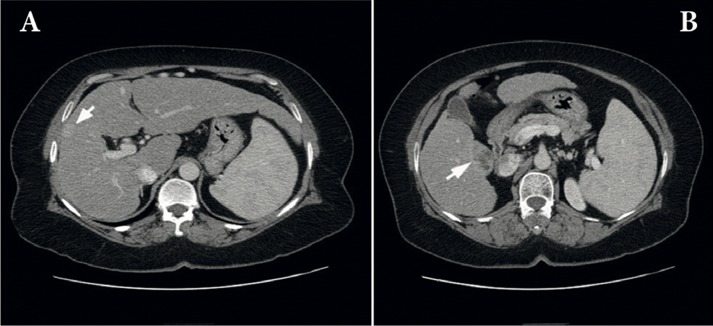
Dynamic Computed Tomography (CT) Before Liver Transplantation A) A 1.5-cm-size focal hepatic lesion with typical vascular pattern of hepatocellular carcinoma (HCC) in segment 8; B) A 3-cm-size focal hepatic lesion with atypical vascular pattern of HCC in segment

**Figure 2:**
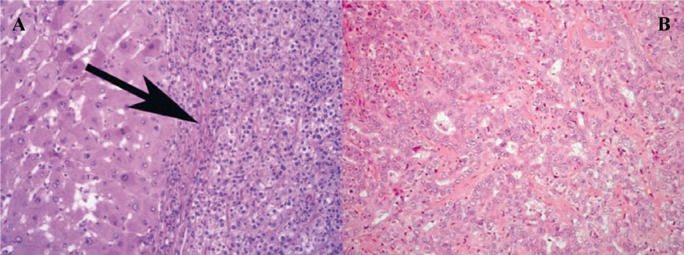
Histopathological Characteristics A) The lesion in segment 8 showing hepatocellular carcinoma; B) The lesion in segment 5 showing cholangiocarcinoma (hematoxylin and eosin [H&E] staining; magnification × 20)

**Figure 3:**
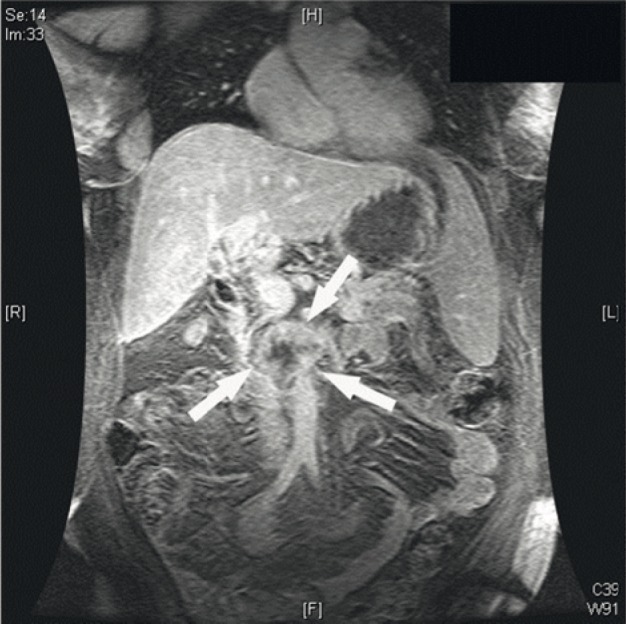
Magnetic Resonance Imaging (MRI) After Liver Transplantation Showing Enlarged Hilar and Paraaortic Lymph Nodes, Which Were Proven to be a Metastatic Cholangiocarcinoma

We report the case of a 56-year-old woman presenting with decompensated cirrhosis caused by hepatitis C virus (Genotype 4). As a part of our routine pre-transplant workup, we performed dynamic abdominal computed tomography (CT), which showed 2 focal hepatic lesions, measuring 1.5 and 3 cm, in the liver segments 8 and 5, respectively. On dynamic imaging, the lesion in segment 8 showed the typical vascular pattern of an HCC (Figure 1A), but that in segment 5 showed an atypical vascular pattern and was closely related to the inferior vena cava (Figure 1B). Serum alpha-fetoprotein (AFP) level was normal, but serum carbohydrate antigen 19-9 (CA19-9) level was slightly elevated (63 U/mL); extrahepatic spread was ruled out. The patient was assumed to have HCC on the basis of the presence of 2 focal hepatic lesions within a cirrhotic liver having viral hepatitis, particularly the smaller lesion in segment 8 that showed the typical vascular pattern of HCC on dynamic imaging. The model for end-stage liver disease (MELD) score was below 14; however, because the patient met the Milan’s criteria for selecting patients with an HCC for LT (6), she was listed for LT and underwent uneventful deceased-donor LT after a 3-month waiting period. Histopathological examination of the explant confirmed that the lesion on segment 8 was an HCC (Figure 2A), but that on segment 5 was a cholangiocarcinoma (Figure 2B). Further histological evaluation of the lesion on segment 5 revealed that the tumor cells expressed cytokeratin (CK) 7, CK19, and carcinoembryonic antigen (CEA) and lacked AFP expression, liver-specific antigens, CK20, and CD34. The cell morphology and phenotype of the tumor cells were consistent with those of cholangiocarcinoma. Our standard immunosuppression protocol includes tacrolimus, mycophenolate mofetil (CellCept), and a tapering course of steroids; however, on the basis of the findings of the 6-week follow-up examination of the explant after LT, tacrolimus was replaced with sirolimus. Three months after LT, we observed a threefold increase in the level of liver enzymes and a viral load of about 9×10^6^ IU/mL. The findings of a liver biopsy revealed the recurrence of hepatitis C virus (HCV) infection. The patient was administered 180 µg of peginterferon alpha-2a (weekly) and 800 mg of ribavirin (daily). Three months later, the level of liver enzymes increased rapidly and the measured viral load was undetectable. A repeat liver biopsy suggested moderately acute cellular rejection. The patient was treated with a 3-day course of pulse steroids, which resulted in a significant decline in the level of liver enzymes. Ultrasonography revealed a mass in the porta hepatis. Serum CA19-9 level was markedly elevated (255 U/mL), and the patient began experiencing vague abdominal pain. MRI confirmed the findings of enlarged hilar and paraaortic lymph nodes that were suggestive of metastases (Figure 3); ultrasound (US)-guided biopsy confirmed cholangiocarcinoma recurrence. Unfortunately, the patient’s functional status deteriorated progressively, and serial chest plain radiographs showed an increase in the extent and number of lung metastases. The patient died from sepsis and respiratory failure 9 months after LT (Table 1).

**Table 1:** Clinical, Radiological, and Laboratory Findings Following the Transplantation

	Before Transplant	Three Months After Transplant	Six Months After Transplant	Nine Months After Transplant
**ALT, U/L**	52	152	475	33
**AST, U/L**	42	171	1590	49
**ALP, U/L**	94	274	682	290
**Bilirubin, µmol/L**	11	10	315	93
**Findings of imaging studies**	2 focal liver lesions	No liver lesions	Mass in the porta hepatis	Mass in the porta hepatis, abdominal lymphadenopathy, and bilateral lung metastases
**Clinical/histopathologi**cal assessment****	Mild ascites	Biopsy was suggestive of HCV recurrence	Biopsy was suggestive of acute cellular rejection	Sepsis, bilateral lung metastases, and respiratory failure
**Alpha-fetoprotein level, ng/mL**	12.7	8.9	4.6	4.4
**Ca19-9, U/mL**	55	65	135	258
**Immunosuppression**	-	Sirolimus, CellCept, prednisone	Pulse steroid, sirolimus, CellCept	Sirolimus

Abbreviations: ALP; alkaline phosphatase, ALT; alanine aminotransferase, AST; aspartate aminotransferase.

## 3. Discussion

Combined HCC and cholangiocarcinoma is a rare form of primary liver cancer that usually presents as "mixed tumors", wherein regions of hepatocellular and biliary epithelial differentiation are intimately mixed within the same tumor (7-10). However, in extremely rare cases, combined HCC and cholangiocarcinoma may present as "collision tumors", wherein the discrete foci of HCC and cholangiocarcinoma arise separately within the liver with no direct contact between the 2 tumors (11-15). Our patient had not been exposed to environmental carcinogens; however, genetic predisposition to developing tumors was not tested. Previous reports have shown poor survival rates and high tumor recurrence rates after LT in patients with cholangiocarcinoma (16). Although LT for cholangiocarcinoma remains controversial, most LT centers do not consider patients with cholangiocarcinoma as suitable candidates for LT (17-20). Cholangiocarcinoma in cirrhotic livers is difficult to diagnose and can be easily mistaken for HCC. Cholangiocarcinoma should always be considered if patients show elevated serum CA19-9 levels and an atypical vascular pattern on dynamic imaging (1, 21); moreover, targeted biopsy or fine-needle aspiration should always be performed in such patients for ruling out cholangiocarcinoma before LT is considered (22). Our patient received a liver from a 27-year-old deceased male donor who was in good health; our patient had an uneventful surgical and postoperative course. Because of positive findings of cytomegalovirus serology in the donor and recipient, the recipient was administered ganciclovir for prophylaxis. Despite the recurrence of HCV infection, followed by an episode of acute cellular rejection, the patient responded well to standard treatment. We believe that the increase in the extent and number of intra-abdominal and pulmonary metastases might have led to sepsis, respiratory failure, and death.

## 4. Conclusions

Collision tumor resulting from combined HCC and cholangiocarcinoma in a cirrhotic liver is rare and has a negative impact on the outcome of LT. Atypical vascular pattern and elevated serum CA19-9 levels are suggestive of such tumors; patients with these findings should undergo a targeted biopsy to rule out the coincidental occurrence of cholangiocarcinoma LT is considered.
